# Mechanisms of Relapse After CD19 CAR T-Cell Therapy for Acute Lymphoblastic Leukemia and Its Prevention and Treatment Strategies

**DOI:** 10.3389/fimmu.2019.02664

**Published:** 2019-11-12

**Authors:** Xinjie Xu, Qihang Sun, Xiaoqian Liang, Zitong Chen, Xiaoli Zhang, Xuan Zhou, Meifang Li, Huilin Tu, Yu Liu, Sanfang Tu, Yuhua Li

**Affiliations:** ^1^Department of Hematology, Zhujiang Hospital, Southern Medical University, Guangzhou, China; ^2^The Second School of Clinical Medicine, Southern Medical University, Guangzhou, China

**Keywords:** CD19 CAR T-cell, relapse, mechanisms, prevention, treatment, strategies

## Abstract

Chimeric antigen receptor (CAR) T-cell therapy is highly effective in the treatment of B-cell acute lymphoblastic leukemia (ALL) or B-cell lymphoma, providing alternative therapeutic options for patients who failed to respond to conventional treatment or relapse. Moreover, it can bridge other therapeutic strategies and greatly improve patient prognosis, with broad applicable prospects. Even so, 30–60% patients relapse after treatment, probably due to persistence of CAR T-cells and escape or downregulation of CD19 antigen, which is a great challenge for disease control. Therefore, understanding the mechanisms that underlie post-CAR relapse and establishing corresponding prevention and treatment strategies is important. Herein, we discuss post-CAR relapse from the aspects of CD19-positive and CD19-negative and provide some reasonable prevention and treatment strategies.

## Introduction

Acute lymphoblastic leukemia (ALL) is one of the most common malignancies in children and adults. Although the 5-year overall survival (OS) rate is 80–90% at present in children (1), relapsed and/or refractory (R/R) ALL remains one of the most important causes of cancer death in children. Chimeric antigen receptor (CAR) T-cell therapy has performed well and has promising applications as an emerging immunotherapy, among which CD19-directed CAR is a remarkable innovation in the treatment of R/R B-ALL. Numerous clinical trials have shown that 70–90% complete remission (CR) can be achieved in pediatric and adult patients treated with CD19-directed CAR T-cells (2–7). However, 30–60% of patients relapse after CAR treatment, and among those, 10–20% are CD19-negative relapse (Table 1); therefore, understanding the mechanisms that underlie relapse is crucial. The eligible treatment options for post-CAR relapse are limited, making it more difficult to achieve CR and improve survival rate; thus, treatments for post-CAR relapse are particularly significant. This article describes the mechanisms of relapse following CAR T-cell therapy and provides some possible strategies for prevention and treatment.

**Table 1:** Summary of published clinical trials of CD19 CAR T cell therapy in patients with ALL.

**References**	**Number of patients (years)**	**Number of patients with pre-HSCT**	**Number of patients bridging to HSCT**	**CAR construct signaling domains**	**Infused cell dose per kg**	**Number of patients with CR (%)**	**Number of patients with relapse (%)**	**Number of patients with CD19+ relapse (%)**	**Median time to CD19+ relapse (months)**	**Number of patients with CD19- relapse (%)**	**Median time to CD19- relapse (months)**
Maude et al. (7)	30 (5–65)	Unknown	Unknown	4-1BB	0.76–20.6 × 10^6^	27 (90)	27 (100)	0	–	4 (15)	~3
Lee et al. 2015 (5)	20 (1–30)	8	10	CD28	1/3 × 10^6^	14 (70)	14 (100)	Unknown	Unknown	2 (14)	~6
Turtle et al. (8)	30 (40–73)	11	13	4-1BB	0.2/2/20 × 10^6^	27 (90)	9 (33)	7 (26)	Unknown	2 (7)	Unknown
Gardner et al. (9)	43 (1–25)	27	11	4-1BB	0.5/1/5/10 × 10^6^	40 (93)	18 (45)	11 (27)	~7	7 (18)	~3
Park et al. (10)	53 (23–74)	19	Unknown	CD28	1/3 × 10^6^	44 (83)	25 (57)	9 (20)	Unknown	4 (9)	Unknown
Maude et al. (11)	75 (3–23)	46	8	4-1BB	0.2–5 × 10^6^	61 (81)	22 (36)	1 (11)	Unknown	15 (25)	Unknown
Li et al. (12)	10 (18–59)	Unknown	Unknown	4-1BB/CD28	0.1–9.79 × 10^6^	6 (60)	5 (83)	5 (83)	~6	0	–
Cao et al. (13)	18 (3–57)	Unknown	4	4-1BB	1 × 10^6^	14 (78)	4 (28)	4 (28)	~1	0	–

*CAR, chimeric antigen receptor; ALL, acute lymphoblastic leukemia; HSCT, haematopoietic stem cell transplantation; CR, complete remission*.

CD19 is a specific B-cell surface marker that plays a crucial role in the differentiation of naive B cells into pre-B cells and maintains the balance of mature B cells in peripheral blood (14–16). Therefore, CD19 is an ideal target of CAR therapy. With genetically modified core receptor CD19 CAR and its recognition independent of major histocompatibility complex (MHC) expression, CD19 CAR T-cells comprise an extracellular CD19 recognition domain, most commonly anti-CD19 single-chain variable fragment (scFv), [FMC63 (CHOP-University of Pennsylvania)/Novartis, NCI/Kite, FHCC-SCRI/Juno-JCAR017] and SJ25C1 (MSKCC/Juno-JCAR015) and signal domain (typically CD3-zeta and costimulatory domain CD28, 41-BB or others). As anti-CD19 scFV binds to antigen on the surface of tumor cells, it can directly activate T-cells and mediate a robust cytotoxic response by releasing cytolytic molecules, perforin, granzyme, and proapoptotic ligand. In addition, activated T-cells can also secrete preinflammatory factors, such as IFN-γ and IL-2, to enhance the immune response.

## Mechanisms of Relapse

There are two patterns of post-CAR relapse in B-ALL, including CD19-positive relapse and CD19-negative relapse. In regard to CD19-positive relapse, whose key mechanism lies in poor persistence of CAR T-cells, CD19 is still present on the surface of B-ALL cells and can be detected by flow cytometry (17). For CD19-negative relapse, CD19 is absent, causing tumors that evade CAR-mediated recognition and clearance in spite of CAR T-cell persistence.

### CD19-Positive Relapse

CD19-positive relapse is usually associated with limited persistence, low potency of CARs, low response to CARs in patients, and transient B-cell aplasia (7).

#### CAR Costimulatory Domain

The CAR costimulatory domain influences the persistence of CAR T-cells. Preclinical studies reported by Zhao et al. (18) showed that 4-1BB costimulatory domain-containing CARs possess greater persistence than those containing a CD28 costimulatory domain. In this experiment, the function and persistence of seven different CAR T-cells with a CD28 or 4-1BB costimulatory domain were investigated. Among them, a configuration utilizing two signaling domains (CD28 and CD3ζ) and the 4-1BB ligand, which also activate the IRF7/IFNβ pathway, presented the highest therapeutic efficacy for tumoricidal activity and T-cell persistence accompanied by an increase in the CD8/CD4 ratio and decreased exhaustion, which supports its higher anti-tumor activity.

#### Source of Single-Chain Variable Fragment (scFv) in CAR

The anti-CD19 scFv used in clinical research is mostly murine-derived, which might result in CAR T-cell exhaustion in patients due to its high antigenicity (13). The binding of murine scFv to the CD19 epitope may trigger the HLA-restricted T-cell-mediated immunoregulatory response, leading to the diminished persistence of CD19 CAR T-cells *in vivo* and even ALL relapse (8), while humanized scFv can reduce the antigenicity of CAR T-cells and enhance its persistence *in vivo* to improve its efficacy.

#### Age-Dependent T-Cell Quality

A report on long-term follow-up of CD19 CAR T-cell therapy in patients with B-ALL (10) suggests that child and young adult patients have longer *event*-free median survival times than adults, implying that the difference may be attributed to age-related changes in the T cells collected from patients.

Kotani et al. (19) compared the function of CAR T-cells derived from young or aged mice, and found that aged CAR T-cells were short-lived and expanded poorly with less memory-like phenotypes in spite of their superior cytotoxicity. While young CAR T-cells were highly active in cell proliferation and cell differentiation. Moreover, Guha et al. (20) examined the differences in CAR expression from geriatric donors and younger donors, with the results showing that CAR T-cells from geriatric donors had significantly lower transduction efficiency and were functionally impaired relative to CAR T-cells from younger donors. The aforementioned researches suggest that differences in clinical outcomes between young and elderly patients might be due to the age dependence of the CAR T-cell phenotype that is reflected by its unique gene expression pattern, secretory profile, and/or transcription factor balance. Therefore, aged CAR T-cells may result in CD19-positive relapse for poor persistence and effectiveness, which might be the reason why child patients achieve longer event-free median survival times (19).

#### Starting T-Cell Phenotype

Starting T-cell phenotype of CAR T-cell manufacturing is crucial to patients' prognosis. Gardner et al. (21) conducted a clinical trial involving 43 children and young adults. Patients treated were assigned to the dysfunctional response group (who obtained early treatment failure) and the functional response group [who obtained a minimal residual disease (MRD)-negative remission that was maintained beyond 63 days]. They detected apheresis-derived starting materials and markers associated with functional exhaustion of domain-containing protein-3 (TIM-3), lymphocyte-activated gene-3 (LAG-3), and cytotoxic T-lymphocyte antigen 4 (CTLA-4) in both groups and found that compared with the functional response groups, the percentage of CD8^+^ T-cells which express LAG-3 and PD-1 in the dysfunctional group was significantly increased. The number of CD4^+^CAR cells and CD8^+^CAR cells in the functional response group at the time of peak implantation was also conspicuously higher. Moreover, in the dysfunctional group, the number of CD8^+^ T-cells expressing TNF-α was less than that in the functional response group, which suggested that an increase in the frequency of cells expressing LAG-3 and a declined ability to secrete cytokines after stimulation can produce CAR T-cell products with reduced anti-leukemic potency, resulting in a CD19-positive relapse.

### CD19-Negative Relapse

Clinical experience has shown that 10–20% of ALL patients developed CD19-negative relapse after CD19 CAR T-cell treatment (Table 1).

#### CD19 Gene Mutation

Recent studies have described that the CD19 gene contains exons 1-13, in which exons 1-4 encode the extracellular domains, and exons 5-13 encode the transmembrane domains. Exon 4 specifically encodes the binding sites of FMC63 in CD19 CARs (22).

Orlando et al. (23) analyzed 12 patients with post-CAR CD19-negative relapse and observed that each patient had at least one distinctive frameshift code insertion or deletion in CD19 exons 2-5, in some cases with missense single nucleotide variants, while recurrence-related mutations were not found in other B-cell antigen genes (including CD22, CD20, CD10, CD34, CD38, and CD45). The study also reported that 8 of the 9 patients acquired loss of heterozygosity (LOH) in CD19 at relapse. The allele frequency (AF) of the mutation is strongly associated with the expected percentage of CD19-negative cells as measured by flow cytometry, which indicates that biallelic frameshift mutations and homozygous loss in CD19 are a major source of CD19 loss and acquired resistance to CD19 CARs.

Alternative splicing is one of the mechanisms leading to CD19 gene mutation. Alternative splicing enriches protein diversity, which plays an important role in normal tissue identity and human growth. However, in tumors, alternative splicing also exists in the aims of evading targeted therapy (24). Similar escape mechanisms have been found in other tumor tissues, such as breast cancer tissue splicing exon 16 of HER2 to escape trastuzumab, and melanoma tissue splicing BRAF (V600E) to achieve dimerization to escape vemurafenib. Studies revealed that there are also mRNA splicing events, particularly in SF3B1, U2AF1, and SRSF2, in hematological tumors such as chronic lymphocytic leukemia (CLL) and myelodysplastic syndrome (MDS) (25).

**Figure 1:**
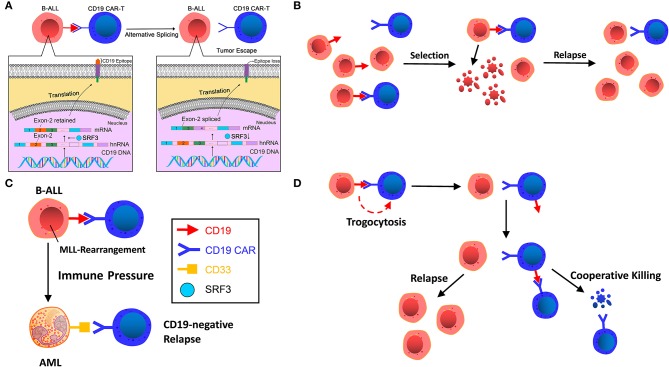
**(A)** Alternative splicing. CD19 gene deletion, frameshift, and exon 2 mutations in patient tumor cell samples resulted in the loss of CD19 epitope recognized by FMC63 of CD19 CAR T-cells. The low level of SRSF3, a splicing factor with its function of retaining exon 2, is the main reason for the loss. **(B)** Selection by immune pressure. A small number of pre-existing CD19-negative tumor cells escape recognition of CD19 CAR T-cells and are transformed to dominant clones under selective therapeutic stress. **(C)** Lineage switch induced by immune pressure. CD19 CAR T-cells induce cell reprogramming and dedifferentiation of B cells or differentiation of non-targeted pre-B cells. **(D)** Trogocytosis and cooperative killing. B-ALL cells change CD19 to CD19 CAR T-cells, resulting in antigen escape and fratricide T cell killing.

In a clinical trial on B-ALL patients with post-CAR relapse, Sotillo et al. (1) observed that CD19 gene deletion, frameshift and exon 2 mutations in patient tumor cell samples resulted in the loss of CD19. They also found that a low level of SRSF3, a splicing factor whose function is to retain exon 2, is the main reason for the loss, thereby lacking the CD19 epitope recognized by FMC63 of CD19 CAR T-cells and producing an N-terminal truncated protein lacking membrane anchoring. Although its function has been retained, circumventing the defects of cell proliferation and B-cell receptor (BCR) signal transduction to some extent, the CD19 variant could not trigger the killing of CD19 CAR T-cells, thus leading to tumor escape (Figure 1A). Jacoby et al. (26) also found the exons 1-3 junctional transcript deletion in the E2a:PBX cell line of mice that acquired CD19-negative ALL relapses via CD19 exon-specific primers, implying the absence of exon 2. Moreover, those mice in the experiment showed earlier relapse compared with the late recurrence caused by lineage switching. Nevertheless, research by Orlando et al. (23) showed that the frequency of alternative splicing was extremely low (0–2.7%) and presented in negligible tumors during screening and relapse. A similar low frequency of exon skips was also found in other genes, suggesting that alternative splicing is a coincidence of CD19 antigen loss, which has yet to be determined by further studies.

#### Selection by Immune Pressure

Generally speaking, the acquired resistance of tumor cells to immunotherapy can be explained by Darwin's theory of natural selection. Before immunotargeting therapy, heterogeneity of the genetics or epigenetic traits of tumor cells already existed. The immunotargeting therapy selects the tumor cells, thereby transforming non-targeted-killing tumor clones into dominant clones and resulting in relapse (Figure 1B).

Grupp et al. (27) performed a flow cytometry test on a pediatric case of CD19-negative relapse following CAR T-cell therapy and found that the small number of pre-existing CD19-negative clones in tumor cells prior to CAR T-cell therapy were transformed to dominant clones under the selective therapeutic stress of CD19 CARs, thus resulting in a CD19-negative relapse.

Intriguingly, Fischer et al. (24) conducted further clinical studies based on the study by Sotillo et al. (1) and obtained different conclusions. Analysis of bone marrow and peripheral blood samples from untreated children and adults with CD19-positive B-ALL and non-leukemia patients revealed that the total deletion (ex2-isoform) and partial deletion of exon 2 (ex2part-isoform) of CD19 in B cells are expressed in both subjects, suggesting that B-cells that cannot be recognized by CAR T-cell therapy for lacking the CD19 epitope might exist in leukemia patients before treatment, even in healthy people. This finding indicates that B-ALL patients can select the CD19 isoform with exon 2 deletion under the stress of CD19 CAR T-cell therapy, rather than alternative splicing of exon 2, without denying the existence of alternative splicing. It also showed that no skips in exons 5 and 6 in cytoplasmic domains and transmembrane expressing CD19 were observed at the beginning of CAR T-cell treatment in children with ALL, suggesting that alternative splicing may occur in exons 5 and 6, causing CAR T-cells to be incapable of recognizing CD19 and thereby causing tumor escape. Nevertheless, further clinical trials on a sufficient number of patients are needed to determine whether the relapse results from alternative splicing or immune selection.

#### Lineage Switch Induced by Immune Pressure

In a murine experiment, Jacoby et al. (26) found that immune pressure rather than immune selection of CD19 by CAR T-cells led to the reprogramming of the B-ALL lineage, resulting in late relapse (Figure 1C). Due to the plasticity of the B-cells' inherent lineage, pre-B-cells can be induced to different phenotypic conversions, including myeloid conversion under immune pressure (2). Late relapse shows the entire loss of CD19 protein and mRNA expression alongside the deletion of major regulators of B-cell development, including B-cell-associated important transcription factors Pax5 and Ebf1, resulting in the loss of B cell development programs. After lineage switch, B-ALL tumor cells undergo phenotypic switch and lead to CD19 expression decline or silencing.

Gardner et al. (2) infused CD19 CAR T-cells into 7 patients with B-ALL harboring gene rearrangement of mixed lineage leukemia (MLL). Two of these patients developed acute myeloid leukemia (AML) relapse and loss of B lymphoid lineage antigens, including CD19, within 1 month, demonstrating that CD19 CAR T-cells could induce myeloid conversion in ALL. The myeloid blasts at relapse might be due to cell reprogramming and dedifferentiation of B lymphocytes or differentiation of non-targeted pre-B lymphocytes, or clonal substitution of pre-existing AML clones in small amounts under the CD19-CAR therapeutic pressure. In addition, the two patients developed severe cytokine release syndrome (CRS), whereas those without a lineage switch did not undergo severe CRS. It has been proved that the severity of CRS is highly associated with IL-6 (28, 29). And IL-6 was found to be a crucial factor driving myeloid differentiation of the (4;11) MLL-B-ALL line (30, 31). Therefore, high cytokine levels in serum during CRS may be conducive to the myeloid differentiation of lymphoid clones and the growth of myeloid leukemia clones. Further analysis of this study (32) affirmed that myeloid conversion rather than immune selection of biphenotypic/bilineal leukemias contributes to relapse. Additionally, KMT2A rearrangement can also lead to lineage conversion (33). The reasons for the lineage switch might be the changed mutation load and tumor microenvironment affected by genomic and epigenetic instability of tumor-switching cells (such as MLL gene rearrangement) under immunotherapeutic pressure (2).

#### Trogocytosis and Cooperative Killing

Trogocytosis (from the ancient Greek trogo, meaning “gnaw”) refers to the phenomenon that lymphocytes can extract some surface molecules from antigen-presenting cells (APCs) through immunological synapses (34). Recently, in the study by Hamieh et al. (35), the researchers marked CD19 of ALL cells using fluorescence marking technology, cocultured ALL cells with CAR T-cells (19-BBζ) *in vitro*, and observed decreased CD19 expression in tumor cells concurrent with a large number of CAR T-cells showed positive CD19 staining, and the transfer of CD19 protein from ALL cells to T-cells showed a characteristic of CAR-mediated trogocytosis. Co-culture of sorted CD19+ CAR T-cells with 19-28ζCAR T-cells caused 19–28ζcells to produce IFNγand GzmB, resulting in killing CD19+ T-cells. This indicates that target antigen transferred by leukemia cells induces resistance to CAR T-cell therapy, also resulting in fratricide T-cell killing (Figure 1D). This finding suggests that CARs provoke invertible antigen loss through trogocytosis, which is an active process in which target antigens are transferred to T-cells, thus reducing tumor cell target density and T-cell activity by facilitating fratricide T-cell killing and T-cell exhaustion.

## Prevention and Treatment Strategies for CD19-Positive Relapse

CD19-positive relapse is of rare occurrence during the persistence of CAR T-cells or continuous B-cell aplasia, whereas CAR T-cell exhaustion and activation-induced CAR T death (AICD) are possible reasons for its short persistence; therefore, improving CAR T-cell persistence is a major strategy for the treatment of CD19-positive relapse.

### Laboratory Strategies

#### Improving CAR Structure

##### Extracellular domain of CAR

The four crucial features of scFv are affinity, immunogenicity specificity and their binding epitopes. Cao et al. (13) performed human-derived CAR T-cells amplification *in vitro* and *in vivo* and found that it had great persistence and killing ability. A clinical trial has demonstrated that after receiving a murine CAR T-cell treatment for B-ALL relapse, infusion of murine CAR T-cells cannot induce CR, whereas infusion of human CAR T-cells (hCART19s) is capable of inducing CR. The trial enrolled 13 patients with R/R ALL who received hCART19s, and 92.9% achieved CR, including the patients with relapse after murine CAR infusion.

##### Transmembrane domain (TM domain) of CAR

The TM domain is the joint between the hinge region and the inner domain of the CAR. Researchers have put type I proteins such as CD3ζ, CD28, and CD8α into use as TM domains in CAR constructs. It was previously believed that the TM domains had little impact on the efficacy of CAR T-cells except anchoring the CAR molecule to the membrane; however, latest studies have suggested that certain specific TM structures contribute to the persistence and anti-tumor efficacy of CAR T-cells. According to Guedan et al. (36), the inducible costimulator (ICOS) TM domain is beneficial for enhancing the persistence and anti-tumor efficacy of the third-generation CARs.

**Figure 2:**
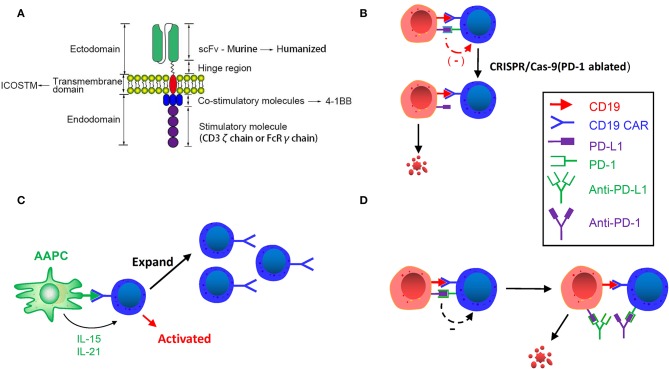
**(A)** Improving CAR structure. Replacing murine scFv with humanized scFv, inducing ICOS TM transmembrane domain and using 4-1BB costimulatory molecule can enhance the persistence of CAR T-cells. **(B)** CRISPR/Cas9 Genome editing in CAR T-cells. Inhibitory receptor co-expression leads to immune cell dysfunction and failure. Using CRISPR/Cas-9 genome editing can downregulate these inhibitory receptors and enhance the activity and persistence of CAR T-cells. **(C)** Designing artificial antigen-presenting cells. Designed artificial antigen-presenting cells release IL-21 and IL-15, and activate CD19 CAR T-cells after remission induction to stimulate and amplify the number of CAR T-cells. **(D)** CAR T-cell binding immunological checkpoint inhibitor. PD-1: PD-L1 initiates T cell programmed death, rendering tumors to gain immune escape. Binding PD-1 and PD-L1 inhibitor can enhance the efficacy and persistence of CAR T-cells.

In a murine experiment, Guedan et al. found that in mice bearing L55 non-small cell lung cancer, CD4 + ICOSz CAR T-cells showed enhanced persistence and then improved persistence. CD8^+^ T-cells expressing either 4-1BB or CD28-based CARs provide evidence that CD8^+^ CAR T-cell persistence is highly dependent on the auxiliary effects provided by intracellular signaling domains (ICDs) for redirecting CD4^+^ T-cells. Additionally, NOD/SCID/gamma (NSG) mice treated with third-generation CAR T-cells that bind ICOS and the 4-1BB signaling domain manifested increased persistence of CD4^+^ and CD8^+^ cells, indicating that ICOS and 4-1BB combined in the third-generation CAR have excellent anti-tumor effects and increased persistence *in vivo*. Intriguingly, the proximal ICD to the membrane has a significant impact on the distal domain in the third-generation CAR. The ideal anti-tumor efficacy and persistence observed in third-generation ICOSBBz CAR T-cells requires ICOS ICD to be located proximal to the cell membrane and linked to the ICOS TM domain. Therefore, CARs with 4-1BB and ICOS ICDs exhibit higher effectiveness than our current 4-1BB-based CARs in the solid tumor models (Figure 2A). The aforementioned description suggests that the TM domain of CAR and its location can have a great influence on the anti-tumor efficacy and persistence of CAR T-cells.

##### Intracellular domain of CAR

Diverse generations of CARs differ in their respective intracellular/costimulatory signaling domains. The first-generation CARs contain merely the CD3ζ intracellular domain, and the second-generation and third-generation of CARs contain the CD3ζ intracellular domain as well as one or two costimulatory domains, such as CD28 and 4-1BB (37). At the time of antigen binding, the phosphorylation cascade of immunoreceptor tyrosine-based activation motifs (ITAM) existent in the intracellular domain of CD3ζ is stimulated to activate CAR T-cells (38). Moreover, the second-generation CAR, which was inserted into the CD28 costimulatory domain on the basis of the first generation, obtained greater expansion and persistence of CARs in the peripheral blood of patients than the first-generation CAR (39).

At the same time, another costimulatory receptor, 4-1BB (CD137), is responsible for enhancing the viability of T-cells. Data show that when using CD28 or 4-1BB CARs to treat patients with ALL, the early response rates are almost similar (3, 5). However, it is different in patients with CLL. According to the research of Brentjens et al., after 19-28z T-cells were infused in 8 CLL patients, a complete reduction was observed in lymph node lesions in 1 patients (12.5%), progressive stability in 3 patients (37.5%), and no objective response in 4 patients (50%). Porter et al. administered the infusion of CTL019 (CD19 scFv + 4-1BB costimulatory domain) CARs to 14 CLL patients. The overall response rate (ORR) was 8/14 (57%), of which 4 patients had CR. The aforementioned two trials demonstrate that 4-1BB CARs exhibit higher efficacy than CD28 CARs in CLL patients, possibly resulting from promoted persistence of 4-1BB (CD137) CARs and depletion of CD28 CARs driven by endothelial cell signaling (4, 40, 41).

#### CRISPR/Cas9 Genome Editing of CAR T-Cell

It is found that regulation of the programmed death ligand 1/programmed cell death 1 (PD-L1/PD-1) axis enhances the anti-tumor activity of T-cells (42, 43). T-cell dysfunction is regulated by the coexpression of negative checkpoint modulators, such as PD-1. Inhibitory receptors, such as LAG-3, TIM-3, and CTLA-4, were also observed in high levels following sustained tumor antigen exposure (44, 45). To date, the functions of these inhibitory receptors can act synergistically to induce immune cell failure (46, 47).

Eyquem et al. (48) reported that targeting a CD19-specific CAR coding sequence to the T-cell receptor (TCR) locus, placing it under the control of endogenous regulatory elements, not only led to uniform CAR expression in human peripheral blood T-cells but also promoted T-cell potency. The *in vivo* performance of the edited cells far exceeds that of the conventional CAR T-cells produced in a mouse model of ALL. Furthermore, targeting CAR to the TRAC locus avoids tonic CAR signaling and establishes effective internalization and re-expression of CAR after single or repeated exposure to antigen, delaying effector T-cell differentiation and failure. The results demonstrate the enormous potential of genome editing for advanced T-cell therapy.

Given the flexibility of genome editing provided by CRISPR/Cas9, it can be used to destroy single or multiple genes encoding inhibitory receptors at the same time (42, 43), thereby “deleting” inhibitory receptors on the surface of CAR T-cells, to increase CAR T-cell persistence (Figure 2B). Effective gene ablation of Fas and PD1 via a one-shot CRISPR protocol has been recently achieved. Furthermore, CD3, HLA-I and Fas triple-negative anti-apoptotic CAR T-cells can be generated by triple gene disruption. The function of Fas-ablated (Fas^neg^) and CD3, HLA-I, Fas triple-ablated (TCR/HLA-I/Fas^neg^) CAR T-cells was examined *in vitro* and *in vivo*. After adding Fas^neg^ CD19 CAR T-cells to K562 leukemia cells expressing the CD19 antigen, reduced apoptosis and increased expression of CARs were detected, and CD3, HLA-I, Fas triple-ablated (TCR/HLA-I/Fas^neg^) CAR T-cells also showed elevated degranulation activity in the experiment, such as CD107 release and enhanced killing ability and cytokine secretion. The prolongation of triple-negative (TCR/HLA-I/Fas^neg^) CAR T-cells in the peripheral blood of CD19 CAR T-cell-treated Nalm6-bearing mice also demonstrated the effectiveness of gene ablation (49).

#### Designing Artificial Antigen-Presenting Cells (AAPCs)

Artificial antigen-presenting cells (AAPCs) were devised to periodically activate CD19 CAR T-cells after remission induction to stimulate and amplify the number of CAR T-cells and prevent antigen-positive relapse. Singh et al. mentioned the usage of K32/4-1BBL AAPC containing the shortened CD19 gene to stimulate amplification of CD19 CAR T-cells (50). AAPCs can also be modified into cells that release specific cytokines. For instance, via transduced IL-21 and IL-15 genes, the APCs would release IL-21 and IL-15 by specific binding, thereby promoting cell proliferation of T-cells and central memory T phenotype cells (Figure 2C) (50–52). AAPCs can also be modified to express an anti-human IgG4 scFv antibody that binds to CAR to allow CAR T-cells to proliferate continuously. More extensively speaking, the use of artificial APCs enhances the efficacy and persistence of infused T-cells (53), providing an idea for addressing the issues of CD19-positive relapses.

### Clinical Strategies

#### Lymphodepletion Regimen

Lymphodepletion regimen refers to chemotherapeutic agents prior to CAR T-cell treatment, reducing tumor burden, eradicating regulatory T-cells to amplify CAR T-cell responses, eliminating other immune cells that may compete for homeostatic cytokines, and enhancing the activation of APCs (54, 55). Current Lymphodepletion regimen of CAR-based therapy mainly include cyclophosphamide (Cy), fludarabine (Flu)/Cy (FC regimen), bendamustine/penitastatin/Cy, Flu/Ara-c (FA regimen), demethylating drug (decitabine)/Cy, etc. Incomplete lymphodepletion may result in limited persistence of CAR T-cells (56). And meta-analysis of Zhang et al. (57) showed that patients who received lymphodepletion regimen before cell infusion achieved a 6-month progression-free survival (PFS) rate of 94.6%, whereas patients who didn't received lymphodepletion regimen only achieved a PFS rate of 54.5% (*p* < 0.001).

In the selection of lymphodepletion regimens, Turtle et al. (8) contrasted the therapeutic outcomes between 17 patients who received FC regimen and 12 patients who received Cy or Cy/etoposide regimen and found that in addition to the significantly increased expansion and persistence of CAR T-cells, there was also improvement in OS and disease-free survival (DFS). Only 2 (12%) of the 17 patients who received the FC regimen relapsed after CAR T-cell infusion, compared with 7(58%) of the 12 patients who did not receive the FC regimen. In the study of Schuster et al. (58), the DFS of the patients receiving FC regimen was also superior to that of patients who received only Cy. Although there is no uniform standard for lymphodepletion regimens currently, the mainstream regimen is the FC regimen.

#### Manufacturing CAR T-Cells With the Central Memory or Stem Cell-Like Memory Phenotype

From the aspect of CAR T-cell manufacturing, starting T cell phenotype has been demonstrated to be an important determinant of subsequent clinical activity. Selection of central memory T-cells (TCM) and stem cell-like memory T-cells (TSCM) can promote continuous proliferation and persistence of T-cells, which are imperative prerequisites for improving therapeutic efficacy. Thus, Blaeschke et al. (59) established a protocol for CD19 CAR T-cell production aimed at high TCM/TSCM quantities. They collected 100 ml of peripheral blood from children with pre-B-ALL, including CD4^+^/CD8^+^ isolation, activation of T-cells with modified anti-CD3/-CD28 reagents and transduction with a 4-1BB-based second-generation CAR lentiviral vectors, and acquired a T-cell product with >100-fold proliferation potential, which was obtained with high functionality and expansion potential and a balanced CD4/CD8 ratio.

Wang et al. (60) conducted two phase 1 studies of central memory-derived CD19 CAR T-cell therapy following autologous hematopoietic stem cell transplantation (HSCT) in patients with B-cell NHL. Adoptive T-cell immunotherapy after HSCT was performed using *in vitro* expanded autologous central memory-enriched T-cells (TCM) transduced with lentiviral transduction expressing CD19-specific CARs. Different T-cell populations and CAR constructs were investigated in two studies, NH1 and NH2. The infusion of engineered TCM-derived CD19 CAR T-cells was performed 2 days after HSCT. In NHL1, 4 of the 8 patients (50%; 95% CI: 16–84%) had no progression at either 1 or 2 years. In NHL2, 6 of 8 patients (75%; 95% CI: 35–97%) had no progression at 1 year. The CD4^+^/CD8^+^ TCM-derived CD19 CAR T-cells (NHL2) showed an increase in expansion. These data demonstrate the feasibility and safety of CD19 CAR TCM treatment after HSCT.

#### CAR T-Cell Binding Immunological Checkpoint Inhibitor

Immune checkpoints are regulatory molecules of inhibition in the immune system that prevent the immune system from producing an effective anti-tumor response. PD-1: PD-L1 initiates programmed death of T-cells, rendering tumors the ability to gain immune escape (Figure 2D) (61–63). In a clinical trial of Maude et al. (64), 6 children with B-ALL received CD19 CAR T-cell therapy in combination with pembrolizumab, 3 of whom showed responses with more durable CAR T-cell persistence. The three responders received pembrolizumab every 3 weeks, and those without responses observed received only one single dose. Analysis of a patient's tumor progression revealed a proliferation of CAR T-cells in the peripheral blood and a decrease in tumor burden. And all patients weren't observed severe CRS. Li et al. (65) reported on 13 pediatric patients with B-ALL relapse who received CD19 CAR T-cell therapy in combination with pembrolizumab. Nine of them have diverse degrees of effectiveness.

Additionally, many clinical trials indicate that the usage of checkpoint inhibitors is of effectiveness and safety in CAR T-cell therapy and can enhance CAR T-cell efficacy and persistence in patients with B-ALL, diffuse large B-cell lymphoma (DLBCL), B-cell non-Hodgkin lymphoma (B-NHL), follicular lymphoma (FL), and so forth (66).

#### Ponatinib Combined With Blinatumomab

For patients with Philadelphia chromosome-positive (Ph+) ALL, tyrosine kinase inhibitor (TKI) is a first-line medication. A phase II trial (67) suggested that 47% of 32 patients with Ph+ ALL who showed resistance to or unacceptable side effects from previous treatment with TKIs such as dasatinib or nilotinib, had responses to Ponatinib. Another study has also demonstrated robust efficacy of Blinatumomab in treating Ph+ ALL even after failure of TKI therapy (68). In a clinical trial, the combination of these two medications has been performed in patients with relapsed/refractory Ph+ ALL, showing safe and effective results (69). Hence, the combination regimen of Ponatinib and Blinatumomab is expected to address the relapse issues after CAR T-cell therapy. El Chaer et al. (70) first reported a patient with Ph+ B-ALL who developed CD19-positive relapse after CD19 CAR T-cells treatment and subsequently responded to the combination of blinatumomab and ponatinib, which is a type of TKI and achieved CR for 12 months, indicating that TKI plays a critical role in the induction and maintenance phases of Ph+ B-ALL therapy. This provides a new approach for the prevention of CD19-positive relapse after CAR T-cell therapy.

## Prevention and Treatment Strategies for CD19-Negative Relapse

### Laboratory Strategies

#### Dual/Multi-Targeted CAR T-Cells

Except CD19, there are a great number of B-cell surface markers, such as CD20, CD22, CD10, CD34, CD38, and CD45, which are also expressed in patients with CD19-positive B-ALL, among which CD123 was widely present on B-ALL cells (71). Dual/multiple-targeted CAR T-cells are a design characterized by combinatorial antigen targeting that simultaneously coexpress two or more different CAR molecules in the same T-cell population. When two or more pan-B-cell markers (CD19 and CD20/CD22/CD123, etc.) are present on the target cells, dual-targeted CAR T-cell activation and robust anti-tumor activity can be triggered in a timely manner. Studies have shown that this method can effectively circumvent lineage switching (26), as well as CD19 antigen loss caused by trogocytosis and synergy (35).

##### Dual-signaling CAR T-cells

**Figure 3:**
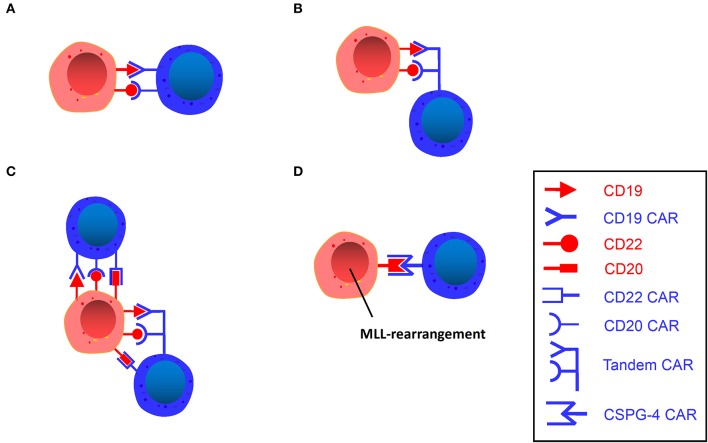
**(A)** Dual-signaling CAR T. A single vector encodes two independent CAR molecules, each recognizing different targets. **(B)** Tandem CAR T. A single vector encodes a bivalent CAR molecule that can recognize two different targets. **(C)** Trivalent CAR T. A single vector encodes three independent CAR molecules or a bivalent CAR molecule plus an independent CAR molecule. **(D)** CSPG4-Specific CAR T. A single vector encodes a CSPG4 CAR molecule that can target MLL-rearranged B-ALL.

A dual-signal CAR T-cell refers to the presentation of two different scFvs in parallel on one T-cell (Figure 3A). Thus far, CD20, CD22, and CD123 are the three most popular targets in studies in addition to CD19. Compared with single-targeted CARs, CD19/CD22 dual-targeted CARs induce more IFN-γ and IL-2 *in vitro* and eradicate patient-derived xenografts (PDX) produced with CD19-negative relapse of CD19-directing CAR treatment (72). Notably, CD123 CAR T-cells were capable of recognizing leukemic blasts, establishing extended synapses, and eradicating CD19-negative leukemia to prolong patient survival, demonstrating that CAR T-cells that bind CD19 and CD123 simultaneously possess superior activity *in vivo* to single-expressing CAR T-cells or combinatorial CAR T-cells against B-ALL (71).

Gardner et al. (73) constructed CD19/22 dual-targeted CAR T-cells through transducing T-cells with two separate lentiviral vectors that direct the expression of two separate CARs targeting CD19 and CD22. This approach produces a mixture of three different CAR T-cell populations (CD19, CD22, and CD19 ×22), which are subsequently performed in 7 patients with R/R ALL (1–26 years old) in phase I clinical trials, among which five achieved CR, and four were MRD-negative. Yang et al. (74) also conducted a phase I clinical trial on 15 patients with R/R ALL, administrating the infusion of CD19 × CD22 dual CAR T-cells. By day 20–30 after CAR infusion, 15/15 (100%) patients achieved CR/incomplete remission (CRi). Eleven patients were bridged into allo-HSCT and have remained in remission state with a median follow up of 133 (97–214) days. Amrolia et al. (75) enrolled 9 patients aged 4–16 years with R/R ALL, and 8 have received CD19 × CD22 dual CAR T-cells. Six of 8 patients achieved MRD-negative CR. Four patients treated at the higher dose of CAR T- cells had an MRD negative CR with ongoing remission, with the longest follow up of 4 months.

##### Tandem CAR (TanCAR) T-cells

TanCAR refers to a tandem arrangement of two different targets on one T-cell (Figure 3B) (76). Bispecific CARs have been devised to recognize two different antigens in a true Boolean OR-gate way (any of the two antigens bound are able to trigger potent T-cell activation) (77). These studies indicate that bispecific CD19-CD20 CARs have demonstrated greater efficacy and less toxicity in preclinical environments than a single CAR under high disease burden. In the experiments of Grada et al. (76), TanCAR T-cells against HER2 and CD19 were cocultured with HER2-positive medulloblastoma cells, CD19-positive lymphoma cells, and breast cancer cells negative for both, with the results showing that TanCAR T-cells only recognized and killed HER2-expressing medulloblastoma cells and CD19-expressing lymphoma cells but not breast cancer cells, which indicates that TanCAR is bispecific to CD19 and HER2 and can induce potent activation of T-cells against single target antigens as well as synergistic enhancement of function upon simultaneous engagement of both.

The antigen-binding domains from FMC63 (anti-CD19) and Leu16 (anti-CD20) antibodies were ligated in different configurations to the transmembrane and T-cell signaling domains to generate tandem CARs. The tandem construct was able to effectively kill CD19-CD20+ leukemia cell lines *in vitro*, and in animals infused with TanCAR 19/20 or 20/19, tumor burden peaked on day 11 and was reduced to below pretreatment levels by day 18, as exhibited by more efficient tumor elimination than the single-targeted CD19 CAR group (>25 days) (78).

In short, TanCAR T-cells reflect the refinement and intelligence of modern immunotherapy, albeit it is only a proof-of-concept design, and the preclinical studies of TanCAR T-cells in animal tumor models have suggested its remarkable application potential in human refractory diseases.

##### Trivalent CAR T-cells

Fousek et al. (79) designed two trivalent CAR T-cell products: one (TriCAR) that expresses three CARs (CD19, CD20, and CD22) separately on the surface of a single T-cell (Figure 3C), and another (SideCAR) that expresses a traditional single CD19 CAR T-cell combined with a TanCAR (CD20 and CD22). This study suggests that TriCAR T and SideCAR T-cells had a more potent killing effect on B-ALL cells than CD19 CAR T-cells. Moreover, bone marrow samples from CD19-negative relapsed patients and CRISPR CD19 knockouts of the three primary ALL samples were specifically obtained in the study, and TriCAR T and SideCAR T-cells could significantly inhibit these cells, while CD19 CARs were ineffective. Further studies revealed that as TriCAR T-cells interacted with CD19 knockout B-ALL cells, the number of immune synaptic (IS) microclusters in different actin polymerization forms of T-cells was significantly elevated, indicating that T-cells have high immune activity after polarization and remodeling, while CD19 CAR T-cells cannot form IS, which demonstrates that trivalent CAR T-cells could effectively alleviate CD19-negative relapse. This strategy may serve as a first-line treatment for primary ALL and as a therapeutic tactic for patients with CD19-negative relapse.

##### CSPG4-specific CAR T-cells

MLL is characterized by a specific translocation of the MLL gene on chromosome 11. As described before, using CD19 CAR T-cells to treat MLL rearranged ALL may lead to susceptible lineage switching. Chondroitin sulfate proteoglycan 4 (CSPG4) (80–84) is discovered on the surface of MLL rearranged leukemia cells, occupying approximately 10% of all leukemias (85). Given the high frequency of recurrence and a significantly decreased OS rate associated with MLL (85) and its enhanced resistance to standard chemotherapy (86), new therapeutic strategies are urgently needed. Therefore, antigen-specific targeting of CSPG4 has attracted increasing attention. Harrer et al. (87) performed an experiment in an attempt to utilize CSPG4-CAR T-cells to treat the target cell line KOPN8, which expresses CSPG4 and has an MLL-MLLT1 translocation of chromosomes 11 and 19 [*t*_(11;19)_]. After coculturing CSPG4-CAR T-cells and KOPN8 leukemia cells, CSPG4-CAR T-cells were activated by KOPN8 cells in an antigen-specific manner, secreting Th1 cytokines and specifically lysing leukemia cells. This experiment demonstrates the potential of CSPG4 as a novel target antigen of CAR T-cells for the treatment of MLL rearranged B-ALL, and therefore, it can serve as a novel therapeutic strategy for patients with MLL rearranged ALL (Figure 3D).

##### BAFF-R-specific CAR T-cells

B cell activating factor receptor (BAFF-R) is a B lineage marker with expression restricted to B cells, which is widely expressed in almost all subtypes of lymphoma and leukemia and is essential for the survival of tumor cells (88–90). Qin et al. (91) developed a humanized second generation CAR T-cell against BAFF-R. In this study, the effects of BAFF-R therapy targeting on a mouse model of B cell malignancies were tested. They found that after treatment with BAFF-R-CAR T-cells, the tumors of the mice regressed significantly and the survival time was prolonged. For relapse, BAFF-R expression was retained in the B-ALL cells with CD19 antigen loss after CD19-directed therapy. By knocking out the CD19 gene of human B-ALL cell lines, BAFF-R-CAR T-cells still exhibited robust efficacy against CD19-negative B-ALL cells whereas CD19 CAR T-cells could not be activated. Subsequently, the researchers compared the effects of CAR T-cells and BAFF-R-CAR T-cells on CD19-negative tumor samples obtained after CD19-targeted therapy (blinatumomab), and found that tumor samples retained BAFF-R expression and the latter was activated and had stronger effectiveness. BAFF-R-CAR T-cell therapy has been demonstrated superiority in multiple comparative trials and it is expected to treat the relapse after CAR T-cell therapy.

### Clinical Strategies

#### Another Single-Targeted CAR T-Cell

Loss of CD19 is a common mechanism of relapse after treatment with CD19-targeted CAR T-cells. Similar to CD19, CD22 is also diffusely expressed in B cells in patients with B-ALL (92–96), and CD22 expression can be detected in a number of patients with CD19-negative relapses (14). Single-targeted CD22 CAR T-cell therapy is also a common therapeutic tactic for CD19-negative relapse. A phase I dose-escalation trial of a novel CD22-CAR with a 4-1BB domain was conducted (97), which enrolled 21 children and adults with R/R B-ALL, involving 17 children who did not receive CD19-directed immunotherapy. A CR rate of 73% was observed in patients receiving CD22-CAR T-cells, involving 5 patients with dim or without expression of CD19 in leukemia cells.

#### Sequential Infusion of Two Groups of Single-Targeted CAR T-Cells

**Figure 4:**
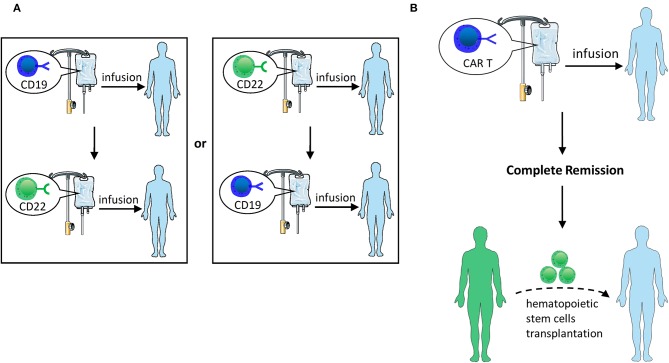
**(A)** Sequential infusion of two groups of single-targeted CAR T-cells. Infuse CD22 CAR T following the infusion of CD19 CAR T or vice versa. **(B)** HSCT after CAR T-cell therapy. After CAR T therapy induces CR, hematopoietic stem cell transplantation can be bridged.

Clinical studies (98) have shown that sequential infusion of third-generation CD19 and CD22 CAR T-cells, which is called cocktail therapy, is feasible and safe for patients with R/R B-ALL (Figure 4A). In a clinical trial, cocktail therapy was used to treat 27 patients with R/R B-ALL. As a consequence, the trial yielded a 6-month OS rate of 79% and an event-free survival rate of 72% with sustained remission, in which 24/27 (88.9%) patients received CR or CRi, and 13/27 (48.1%) patients attained MRD-negative CR. The center subsequently enrolled more candidates (99), among whom 81 patients received CAR22 T-cells following the infusion of CD19 CARs, while 8 patients received CD19 CARs following the infusion of CD22 CARs. The median follow-up time was 7.6 months. Among 50 evaluable patients, 48 (96.0%) achieved CR/CRi by day 30, 94% of whom were MRD-negative. The PFS of B-ALL patients was 12.0 months, and the median OS was not reached. In total, 23 patients experienced a relapse, with no CD19 or CD22 antigen loss observed. Drawing on the finding that a high MRD-negative rate in R/R ALL patients was achieved by sequential infusion of third-generation CD22 and CD19 CAR T-cells, demonstrating this method has great feasibility for the treatment of CD19-negative relapse ALL.

#### HSCT After CAR T-Cell Therapy

HSCT is currently recognized as the only potential treatment for all R/R patients who achieve CR (100, 101). Although CAR T-cell therapy could induce a high CR rate, it still often results in recurrence. HSCT following CR with CAR therapy is probably feasible and viable to cure R/R ALL (Figure 4B). Current studies suggest that CAR T-cell therapy may serve as a bridge treatment regimen prior to HSCT, providing patients with more opportunities to receive treatment and potentially improving the overall outcome of these patients (5, 102, 103). Clinical studies by Haneen (104) showed that CAR T-cell therapy followed by HSCT could synergistically improve leukemia survival. Lee et al. (5) reported that 10 children with R/R ALL who proceeded to allo-HSCT after entering MRD-negative CR with CAR T-cell infusion retained consistent remission without subsequent recurrence. Brentjens et al. (3) reported 4 patients who received allo-HSCT after CAR T-cell therapy and retained MRD-negative CR for 3–6 months. Yang et al. (74) reported that 19 patients were administered dual-target CAR T-cell therapy, and 18 of them entered CR. Among those patients who achieved CR, 14 patients subsequently proceeded to allo-HSCT, with a median OS of 236 days and a PFS of 234 days. There were no recurrences in patients who underwent CAR therapy bridging allo-HSCT, and 3 of 4 non-transplanted patients relapsed. Gardner et al. (2) also suggested that HSCT should be performed as soon as possible to avoid myeloid conversion after entering CR by CAR T-cell therapy.

Nevertheless, whether allo-HSCT should be performed after CAR T-cell-induced remission of R/R ALL remains controversial. Davila et al. (103) reported that 7 of 16 patients with R/R ALL proceeded to allo-HSCT following CAR T-cell therapy, with encouraging outcomes in that none of them experienced relapse. In another study (105) of 37 adults with R/R ALL who underwent CD19 CAR T-cell therapy, 13 of them proceeded to allo-HSCT after achieving CR. Compared to those who did not undergo allo-HSCT, there was no significant difference in 6-month OS (79% vs. 80%). The inability to detect statistically significant differences may be due to the short duration of follow-up and the insufficient number of cases. Despite this, CAR T-cell therapy bridging to allo-HSCT still has considerable therapeutic potential, and it requires the support of large numbers of samples in forthcoming clinical trials.

## Discussion

CD19 CAR T-cell therapy is among the most promising therapies for B-cell malignancies, but relapse has emerged as a great threat to patients. With regard to CD19-positive relapse, the focus should be CAR T-cell persistence. However, for CD19-negative relapse, the mechanisms are not fully elucidated; for instance, alternative splicing is still controversial and requires further exploration. Moreover, the mechanisms are relatively fragmented and weakly correlated, which means that it is particularly difficult for us to establish an optimal curative paradigm to address the recurrence issue with one single treatment. Therefore, different schemes from these two aspects regarding post-CAR relapse were formulated. For those with CD19-positive relapse, efforts should be made in CAR T-cell persistence improvement to improve therapeutic efficacy and decrease the risk of relapse. In the clinic, the efficacy of CAR-based therapy is associated with a quantity of parameters. With the update of CAR T-cells generations and the application of fully humanized scFv, the persistence of CARs has been greatly improved. In addition to using improved humanized CARs, it should be focused on T-cell quality and whether effective T-cells could be extracted. After treated with large doses of drugs or underwent repeated chemotherapy, the quantity and quality of T cells *in vivo* are affected. Therefore, CAR T-cells generated from healthy donor T-cells may be one feasible therapeutic direction, bringing benefits of lower cost, higher availability (106) and anti-tumor effect (107). For CD19-negative relapse, the underlying mechanisms vary with weak correlations, presenting a set of challenges for CAR T-cell treatment. In this review, we have provided corresponding strategies for the prevention and treatment of both resistance mechanisms that underlie relapse, such as dual/multi-targeted CAR T-cells for lineage switch, natural selection, trogocytosis, and synergy; CSPG4-specific CAR T-cells for lineage switch; and other clinical strategies. Although various treatment strategies are emerging at present, associated problems have been gradually exposed. For instance, although multiple antigens are targeted, there may still be a small number of pre-existing clones that hard to kill and a lineage switch resulting from immune stress. Furthermore, the high preparation difficulty, economic factors and multiple target issues complicate the clinical analysis and strengthen the heterogeneity and antigenicity of the infusion product, which easily causes adverse reactions, leading to a robust immune response *in vivo* and the declining persistence of CARs. In CAR-based therapy, antigen is the essential issue and the major determinant affecting the efficacy. It is well-recommended that conducting antigen and even gene detection before CAR treatment and after relapse and choosing different CAR T-cells regimens in accordance with specific conditions of patients.

At present, attempts to overcome the limitations of CAR T-cell therapy mainly concentrate on the improvement of safety and efficacy. Based on the mechanisms of relapse, it is likely to treat ALL relapse more effectively. Hopefully, emerging novel therapeutics will be brought into the clinic as soon as possible and provide patients with more options. As CAR T-cell technology continues to develop, it is also expected that emerging technology can be carried out in the clinic soon to provide patients with more curative options, and to specific-targeted and precise treatments will come true once it surpasses the limitations of technology and economic benefits. Thus far, a substantial number of studies on CAR-based treatment have been conducted, but large-scale clinical trials on CAR T-cell therapies are still scant and efforts to explore further are underway. Confirming the efficacy of relatively mature CAR T-cell therapy remains an active area of forthcoming clinical trials. On many occasions, our treatments rely on clinical experience and lack evidence-based support to establish therapeutic strategies. Therefore, it is hoped that a standardized clinical guideline for CAR T-cell treatment after enough prospective clinical studies will be formulated, and in the near future, CAR T-cell therapy will be more optimized and established so that the patients' prognosis can be upgraded to a new level.

## Author Contributions

This study was conceived and designed by ST and YLi. Screening of papers and data extraction was performed by XX. Writing of the first draft of the manuscript was performed by XX, QS, and XL. ZC, XZha, XZho, ML, HT, and YLiu contributed to the writing and revision of the manuscript. All authors approved the final version of the manuscript.

### Conflict of Interest

The authors declare that the research was conducted in the absence of any commercial or financial relationships that could be construed as a potential conflict of interest.
